# Inequality in the distribution of ^137^Cs contamination within freshwater fish bodies and its affecting factors

**DOI:** 10.1038/s41598-021-85291-6

**Published:** 2021-03-11

**Authors:** Nobuyoshi Ishii, Toshio Furota, Maiko Kagami, Keiko Tagami, Shigeo Uchida

**Affiliations:** 1 National Institutes for Quantum and Radiological Science and Technology, Environmental Transfer Parameter Research Group, 4-9-1 Anagawa, Inage-ku, Chiba, 263-8555 Japan; 2 Faculty of Science, Department of Environmental Science, Toho University, 2-2-1 Miyama, Funabashi-shi, Chiba, 274-8510 Japan; 3 Graduate School of Environmental and Information Sciences, Yokohama National University, 79-1 Tokiwadai, Hodogaya-ku, Yokohama, 240-8501 Japan; 4 National Institutes for Quantum and Radiological Science and Technology, Biospheric Assessment for Waste Disposal Team, 4-9-1 Anagawa, Inage-ku, Chiba, 263-8555 Japan

**Keywords:** Environmental sciences, Limnology

## Abstract

Contamination of freshwater fishes with ^137^Cs remains as a serious problem in Japan, nearly 10 years after the Fukushima nuclear power plant accident, but there is limited information on the distribution of ^137^Cs contamination in fish bodies. The ^137^Cs distribution can be used for the estimation of internal radiation exposure through the consumption of fish and for the dose estimation of fish themselves. In this study, the ^137^Cs distribution in the bodies of 8 freshwater fish species was investigated as percentages of total body burden for fish inhabiting Lake Inba. Fish samples were caught in stake nets placed close to the shore approximately once a month. After the measurement of body length and fresh weight, the radioactivities of ^137^Cs in muscle, internal organs, spawn, milt and bone were assayed using high-purity germanium detectors. Analysis of all fish samples showed that the ^137^Cs distribution was highest in muscle (54 ± 12%), followed by internal organs (7.8 ± 4.6%), spawn (7.4 ± 5.4%), milt (3.2 ± 2.1%) and bone (1.2 ± 0.58%). Among fish species, the highest proportion of ^137^Cs in muscle was detected in largemouth bass (71 ± 1 3%), followed by snakehead (69 ± 14%), channel catfish (63 ± 17%), common carp (62 ± 14%), barbel steed (58 ± 6.5%), silver carp (57 ± 7.7%), bluegill (53 ± 4.7%), and crucian carp (50 ± 10%). These results suggested that the ^137^Cs in muscle was likely to be high in piscivorous fishes compared to omnivorous fishes, especially crucian carp. The proportion of ^137^Cs in muscle of crucian carp was not explained either by body length or fresh weight. However, a positive correlation was found between the proportion of ^137^Cs in muscle and the condition factor which was an indicator of nutritional status calculated from a length–weight relationship. This correlation implied that more ^137^Cs accumulated in muscle tissue of a fish species with high nutritional status. This is the first study to show that condition factor is more important than body length and wet weight in explaining the high proportion of ^137^Cs in muscle tissues, at least for crucian carp.

## Introduction

A large amount of radiocesium (^137^Cs + ^134^Cs) was released into terrestrial and marine environments following the Fukushima Dai-ichi Nuclear Power Plant (FDNPP) accident in 2011^1^. Consequently, freshwater environments are contaminated, and freshwater fish have accumulated radiocesium into their bodies^2–4^. The activity concentrations of ^137^Cs in freshwater fishes are relatively high compared to those in marine fishes due to the balance of osmoregulation and excretion of hypotonic urine^5^. Even now, more than 9 years after the accident, some of freshwater fish still contains a higher concentration of total radiocesium than the Japanese standard limit for general foods of 100 Bq kg^−1^ which have been in force since April 1, 2012. In fact, government restrictions and self-imposed restrictions of distribution for freshwater fish have been continued in some regions in Japan. Fish is one of the sources of dietary protein, and the importance of inland water fisheries has globally increased^6^. This raises concerns about internal exposure of humans through consumption of contaminated freshwater fish.

Food cultures have developed all over the world, and edible parts and cooking methods differ among localities. The common edible part of most fish is muscle, and thus muscle tissue is often selected for the measurement of radiocesium activity concentrations^7^. There is also a custom, however, to eat a whole fish, without removing the bones, skins, internal organs and so on. Because the internal radiation dose of humans is influenced by their dietary habits, information on the distribution of radiocesium in fish bodies is useful for improving the internal dose estimation.

There is some interest in environmental protection from ionizing radiation due to the FDNPP accident. The ICRP has said that the purpose of the radiation protection of the environment is to maintain biological diversity, to conserve species, or to keep the health and status of natural habitats, communities, and ecosystems^8^. In order to achieve this purpose, the estimation of dose–response relationship is essential, and this relationship is built on dose estimation of environmental organisms. In most cases, fish muscle tissue is selected for the measurement of radiocesium activity concentrations because of its consumption by the people^7^, but a homogeneous distribution of radiocesium within a fish body is unlikely. Therefore, data on distribution of radiocesium in various tissues and organs will also provide valuable information regarding dose estimation of freshwater fish species.

Lake Inba in Chiba Prefecture, Japan, was contaminated with radioactive fallout from the FDNPP accident^9^. Fish living in Lake Inba have been mainly contaminated with ^137^Cs released by the accident. In this study, the radioactivity concentrations of ^137^Cs were measured in muscle, bone, internal organ, spawn, and milt of 8 kinds of freshwater fishes which were caught in Lake Inba. The amounts of ^137^Cs in each tissue and organ were calculated from the activity concentrations of ^137^Cs and weights of tissues and organs, and then the distributions of ^137^Cs were determined as the ratio of ^137^Cs amount in each part relative to its amount in the whole body. In addition, factors influencing the ^137^Cs distribution were discussed.

## Results and discussion

### Status of ^137^Cs contamination in Lake Inba

Since all the fish samples were from Lake Inba, status of ^137^Cs contamination in this lake is described first. Water from Lake Inba is used as raw water for drinking-water, and the quality is of a concern to local residents. The Ministry of the Environment (MOE) has investigated the activity concentration of ^137^Cs in water of this lake^10^. Since the ministry’s investigation started, ^137^Cs concentrations in the water column have all been below the screening levels for drinking-water for gross beta activity, 1 Bq L^−1^^11^. The MOE results gave arithmetical mean values of the concentration as 0.029 ± 0.012 Bq L^−1^ in the surface (top 0.1 m) and 0.031 ± 0.017 Bq L^−1^ in the bottom (0.5 m from the surface) layers. It should be noted that these values of ^137^Cs were obtained from unfiltered water of the surface and bottom layers. Lake Inba is shallow, and mixing of the water body by winds can cause almost the same concentration of ^137^Cs to be reached in both layers. Although the sediment in Lake Inba is a sink for ^137^Cs^12^, there might be little elution of ^137^Cs from the sediment to the water column. Little variation in the ^137^Cs concentrations after 2015 suggested that fish caught in this study had been living under an equilibrium or an apparent equilibrium condition for ^137^Cs in the water column.

### Distribution of ^137^Cs in freshwater fishes

**Figure 1:**
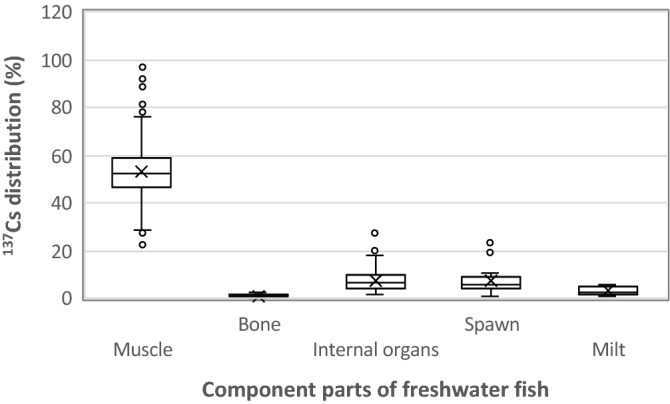
Boxplot showing the distribution of ^137^Cs in muscle (n = 308), bone (n = 28), internal organs (n = 94), spawn (n = 38) and milt (n = 7) of all freshwater fishes caught in this study. Each box indicates the inter-quartile range. The line inside the box shows the median. Marks “×” and “open circles” are mean values and outliers, respectively. Significant difference was found for the Kruskal–Wallis test (*P* < 0.01). The Steel–Dwass post-hoc test showed significant differences except between bone and milt, internal organ and spawn, and spawn and milt.

Distributions of ^137^Cs were significantly different among tissues and organs (Fig. 1, *P* < 0.01). The Steel–Dwass post-hoc test showed significant differences except between bone and milt, internal organs and spawn, and spawn and milt. The highest distribution of ^137^Cs was found in muscle. The arithmetic mean values of the distribution were decreased in the order of muscle tissue (54 ± 12%), internal organs (7.8 ± 4.6%), spawn (7.4 ± 5.4%), milt (3.2 ± 2.1%) and bone (1.2 ± 0.58%). The highest concentration of ^137^Cs or stable Cs being in muscle tissue has also been observed in various organisms such as humans^13^, mice^14^, rats^15^, cattle^16^ and wild boars^17^. Accumulation of ^137^Cs in muscle seems reasonably common in fish and other animals. Although the sum of values for the distribution was less than 100%, the remaining ^137^Cs, approximately 26%, might be distributed to other parts such as the head, gills, skin and fins.

Although the proportion of ^137^Cs in milt was low at 3.2% of the total body burden, the activity concentration of ^137^Cs in this organ was found to be 1.5 times higher on average compared to the whole body. Saxén and Koskelainen^18^ reported that the activity concentration of ^137^Cs in sperm of gutted fish was higher than that in the whole fish, but this finding was based on limited data (n = 5). The present study also had a similar result, and the relatively small value of the ^137^Cs distribution in milt (3.2%) must have been due to the small weight of milt compared to body weight. Since germ cells are sensitive to ionizing radiations^19^, accumulation of ^137^Cs in milt may affect reproduction. This study, however, examined only 7 milt samples, and detailed studies on the distribution and accumulation of ^137^Cs in milt are required in heavily contaminated areas.

**Figure 2:**
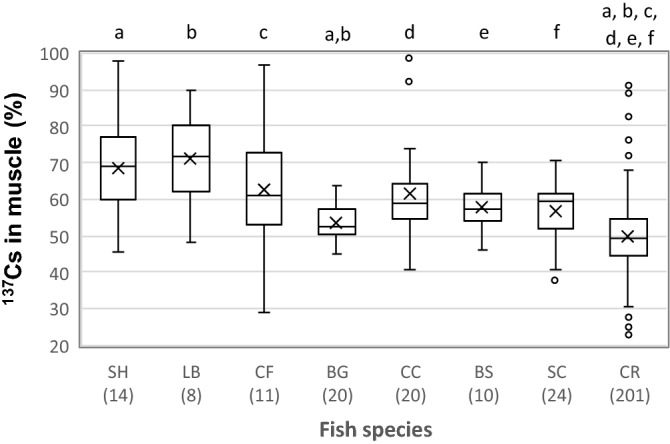
Boxplot showing the proportion of ^137^Cs in muscle of 8 kinds of fishes. Abbreviations of SH, LB, CF, BG, CC, BS, SR, and CR are snakehead, largemouth bass, channel catfish, bluegill, common carp, barbel steed, silver carp, and crucian carp, respectively. The numbers in parentheses are the number of samples. Significant difference was found for the Kruskal–Wallis test (*P* < 0.01). Lowercase letters above boxes indicate significant differences from the post-hoc Steel–Dwass test.

Physiology and ecology of fish vary by species, and thus differences in fish species may affect the level of the ^137^Cs distribution. Since it was shown that ^137^Cs taken in by all freshwater fishes studied was mainly distributed in muscle (Fig. 1), levels of the ^137^Cs distribution in muscle were compared among the species (Fig. 2). A significant difference was found (*p* < 0.01). The highest mean value was 71 ± 13% for largemouth bass, and then the level decreased in the order of 69 ± 14% for snakehead, 63 ± 17% for channel catfish, 62 ± 14% for common carp, 58 ± 6.5% for barbel steed, 57 ± 7.7% for silver carp, 53 ± 4.7% for bluegill, and 50 ± 10% for crucian carp. Similar results have been reported for perch and pike from lakes in Finland, and the distribution of ^137^Cs in muscle of these fish species were 68%-75%. The proportion of ^137^Cs in muscle of crucian carp was significantly lower than that of all other species except bluegill (Steel–Dwass’s test, *p* < 0.05). Similarly, the ^137^Cs distribution level for bluegill was significantly lower compared with levels for largemouth bass and snakehead, but no differences were observed among other species.

These differences in the ^137^Cs distribution among fish species can be explained by food habits. Trophic levels (TLs) of bluegill and crucian carp are 3.2 and 3.1, respectively (FishBase, http://www.fishbase.org), and these values are lower than those of snakehead (TL = 4.4) and largemouth bass (TL = 3.8). Because differences in trophic levels mean differences in food habits, these results suggest that food habits affect the proportion of ^137^Cs in muscle. In addition, piscivorous fish muscle is known to have a higher activity concentration of ^137^Cs compared to non-piscivorous fish muscle^20–22^, suggesting the former easily accumulate ^137^Cs in muscle tissue. Food habits of fish change during their growth process. For example, juvenile snakehead mainly consume zooplankton, and the mature feed on insects, shrimps and fish^23^. Although there was no direct evidence for the difference in food habits between omnivorous bluegill and crucian carp and piscivorous snakehead and largemouth bass in this study, their dietary foods might have been different judging from their body size and physical characteristics. The minimum body length of snakehead, *Channa striata*, at maturation is estimated to be from 17 to 19 cm^24^. In this study, the minimum body length was 24.5 cm. Thus, all of the snakehead caught in Lake Inba must have been mature although it is necessary to consider differences in habitat and species. Mature snakehead feed mostly on other fish, frogs, crustaceans and some insects^25^. Similarly, the minimum body length of largemouth bass was 25.2 cm (29.4 cm of total length) in this study, and this largemouth bass was holding eggs. Paragamian^26^ reported that largemouth bass with a total length of above 20 cm consumed smaller fish more frequently. On the other hand, crucian carp are omnivorous. *C. auratus langsdorfii*, a kind of crucian carp, feed on plant fragments and zooplankton^27^, and *C. cuvieri,* also a kind of crucian carp, have pharyngeal teeth which are adaptive for phytoplankton feeding^28^.

The digestive system might also play an important role as a factor affecting the ^137^Cs distribution in fish bodies. A carnivorous digestive system differs from an omnivorous one. It is known that intestine lengths of omnivorous fish are longer than carnivorous fish^29,30^. The longer digestive tract is thought to have a higher surface area and allow a longer retention time of the food^31^. Consequently, the longer digestive system is useful for enhancing nutrient absorption from indigestible food. The intestines of omnivorous and herbivorous fishes often exhibit higher carbohydrase activities to digest lower quality food such as fiber-rich diets^32^, whereas abilities to digest more easily digestible food such as protein-rich diets are low compared to carnivore fish^33^. The proteins are the most important muscle constituents^34^, and efficient digestion of muscle by carnivore fish cause efficient nutrient and mineral absorption. Muscle also contains a constant level of potassium^35^, and this mineral has important physiological roles^36,37^. Therefore, potassium is an essential element for muscle function. Potassium in muscle is more efficiently absorbed by piscivorous fish than omnivorous fish, and it is immediately distributed to the fish's own muscle tissue. Similarly, ^137^Cs which is a homologous element of potassium may be absorbed and distributed to muscle tissue through digestion. This may be the reason that the difference in digestive system causes differences in the distribution of ^137^Cs among species.

### Factors affecting the proportion of ^137^Cs in muscle

Wide range values of the proportion of ^137^Cs in muscle were observed for crucian carp (Fig. 2, 22.8–91.2%). To determine the factors affecting the proportion of ^137^Cs in muscle, effects of season, length of exposure time, and physical characteristics (body length, fresh weight, and condition factor) on the ^137^Cs proportion were investigated.

**Figure 3:**
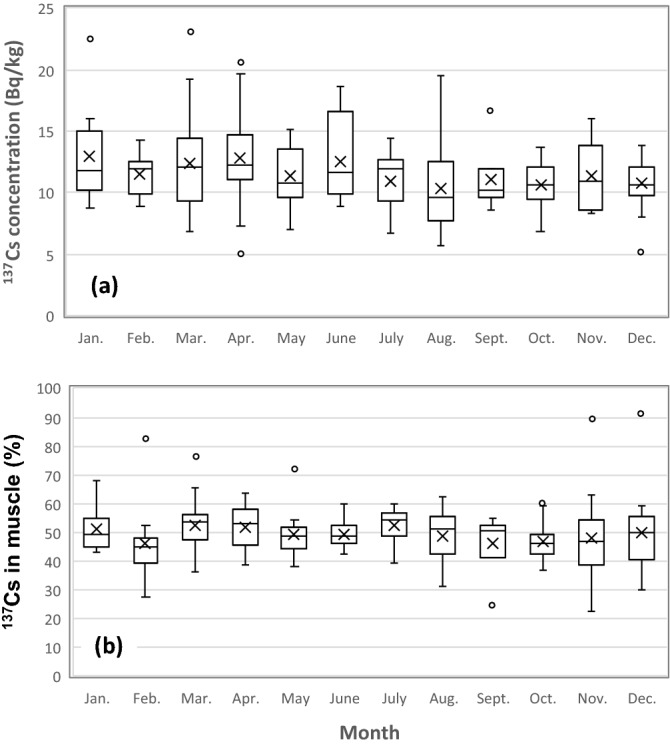
Boxplots showing the seasonal variations of the ^137^Cs activity concentrations (**a**) and the proportion of ^137^Cs in the muscle tissues of crucian carp (**b**) which were caught each month and assayed. Both the concentration and distribution had no difference.

The activity concentrations of radionuclides in the muscle tissues vary seasonally for wild animals such as roe deer^38,39^, wild boar^38,40^, and black bear^40^, and it is considered that changes in food diets of these animals caused the seasonal variation. However, limited data are available for the seasonal variation in the ^137^Cs concentration and distribution of freshwater fish. To fill this gap, both the activity concentration of ^137^Cs and the proportion of ^137^Cs in muscle tissue were compared among the population subgroups of crucian carp caught each month (Fig. 3). The results showed no differences for both the concentration and the distribution. Although these data included values of both male and female crucian carp, different results may obtain when using only female data. Eggs of crucian carp develop from autumn to spring in Lake Inba, and the proportion of ^137^Cs in muscle may decrease during the incubation period by a dilution effect. In addition, a decrease in the ^137^Cs proportion was also expected after the spawning. However, no seasonal variation of the proportion of ^137^Cs even in female muscle was observed (Fig. S2). These results suggested that seasonal changes in physiology, food habits and spawning did not affect the proportion of ^137^Cs in muscle for crucian carp inhabiting Lake Inba.

**Figure 4:**
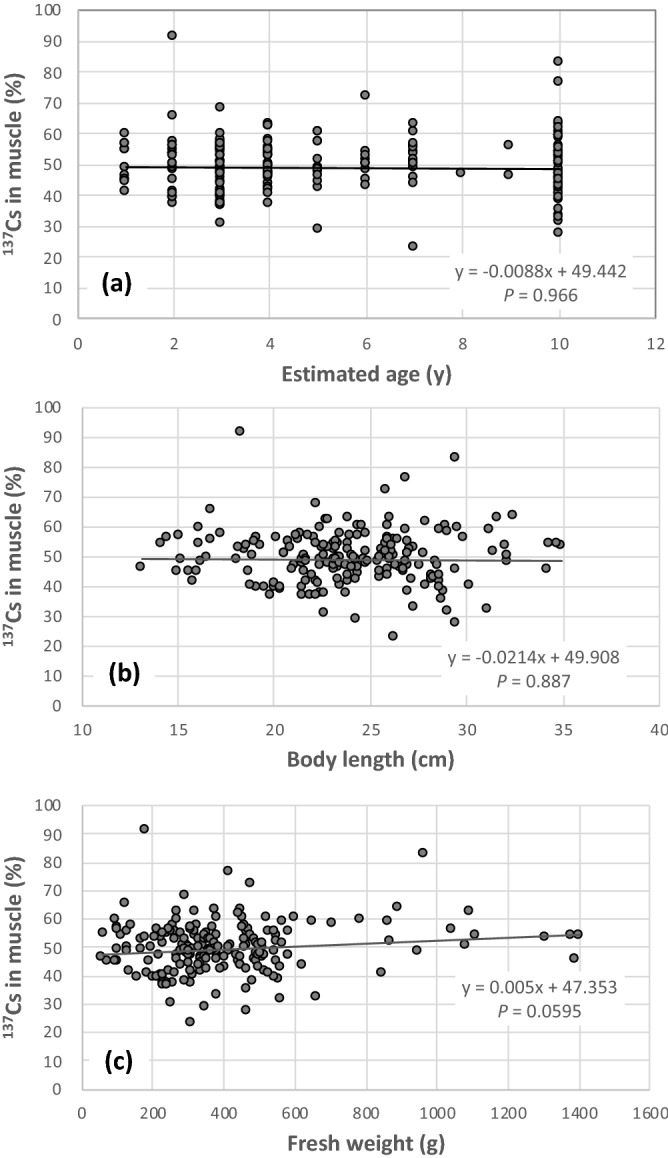
Relationships between the indexes of the growth and the proportion of ^137^Cs in muscle of crucian carp. The estimated (**a**) age, (**b**) body length, and (**c**) fresh weight were used as the indexes. The number of samples was 197. No correlations were observed for any indexes.

The effects of the length of exposure time and body size (length and weight) on the proportion of ^137^Cs in muscle tissue were examined. The length of exposure time is known to affect the accumulation of heavy metals by fish^41^. Together with length of exposure time, fish size must also have an effect, since fish grow over time. In fact, the activity concentration of ^137^Cs in muscle increases with fish weight^42^ and total length^43^, and this is known as ‘size effect’. In this study, estimated age, body length, and fresh weight were used as indexes of the time of exposure. The estimated ages were determined from the growth curve of crucian carp in Lake Inba. The relationships between these three indexes and the proportion of ^137^Cs in muscle of crucian carp are shown in Fig. 4. No correlations were observed for any indexes. This might be partly because crucian carp are not predatory, and no size effect for ^137^Cs accumulation has been found in predatory fish^44^. In addition, the individuals examined were large (> 13.1 cm in body length), and they must have undergone the ontogenetic diet shift. Food and feeding habits of freshwater fish shift ontogenically^23,45,46^, and the major diet component of crucian carp changed changes from plankton to insects with increasing body size^47^. Changes in food habits occur in individuals at a body length of approximately 2 cm^48^, which was much smaller than the examined individuals (> 13.1 cm in body length). Furthermore, crucian carp examined in this study include both *C. auratus langsdorfii* and *C. cuvieri*. Despite being the same genus, food habits of *C. auratus langsdorfii* and *C. cuvieri* are different, which might have caused unclear relationships between the ^137^Cs distribution and size.

**Figure 5:**
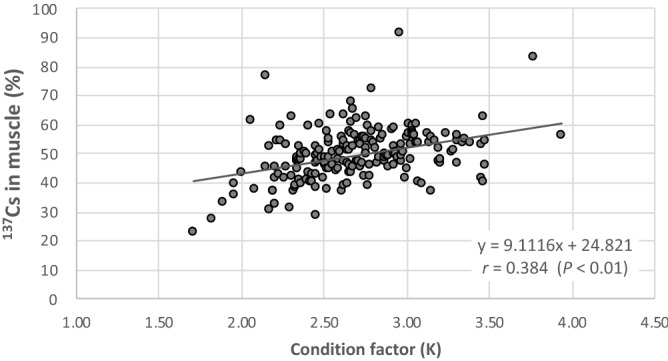
Relationship between the condition factor and the proportion of ^137^Cs in muscle of crucian carp. Positive correlation was observed between the two variables (n = 197).

Finally, the condition factor, which is an indicator of nutritional status, was found to influence ^137^Cs distribution (Fig. 5). Even if fish species live in the same environment, nutritional status varies from individual to individual. However, there are few data on the relationship between nutritional status and the ^137^Cs distribution. Based on the assumption that a heavier fish of a given length is in better condition^49^, the condition factor expressed as the ratio of total body weight and the cube of body length is often used as the indicator of nutritional status and relative health of fish. Therefore, the condition factor was used as a way to estimate nutritional status, and the relationship between condition factor and the proportion of ^137^Cs in muscle of crucian carp was determined (Fig. 5). Spearman’s correlation coefficient showed a positive correlation between the two variables (*P* < 0.01). When the data for the condition factor were divided into quartiles, it was found that the population classified into the first quartile (*Q*_1_) had a lower ^137^Cs distribution than the other three populations (Fig. S3). The condition factor is known to correlate positively to total lipid content of fish^50^. That is, fish with low condition factor values have small amounts of lipids in their muscle tissues. Fats which are a type of lipid are not a reservoir for ^137^Cs in wild boar^51^. Although there are differences in fat components between wild boar and fish, the accumulation of ^137^Cs in fish lipids can be considered as hard to achieve. The fact that a small amount of ^137^Cs was distributed in muscle despite the low amount of lipids means that it is hard for ^137^Cs to accumulate in muscle tissue of fish with poor nutritional status. Since crucian carp were caught in a certain area during the entire study period, there would not be much difference in the quality of the food. Quantity of food intake, therefore, is probably an important factor affecting the proportion of ^137^Cs in muscle at least for crucian carp in Lake Inba.

## Conclusions

This is the first study to document that nutritional status of fish is an important factor affecting the proportion of ^137^Cs in muscle tissue in addition to differences in fish species and trophic level. Previously there were only limited data on the distribution of ^137^Cs in each part of a fish body compared to data on activity concentrations of ^137^Cs in fish muscle. In this study, the ^137^Cs distribution within freshwater fish bodies were investigated in Lake Inba. The obtained results showed that the ^137^Cs distribution differed among body parts. The highest proportion was found in muscle, and the proportion of ^137^Cs decreased in the order of internal organs, spawn, milt, and bone. Moreover, the proportion of ^137^Cs in muscle differed among fish species. Fish with high trophic levels easily distributed more ^137^Cs in their muscle tissue than fish with low trophic levels. To determine factors affecting the ^137^Cs distribution in muscle, the effects of season, time of exposure and physical characteristics on the proportion of ^137^Cs in muscle were investigated using crucian carp. Only the condition factor as an indicator of nutritional status affected the ^137^Cs distribution. Fish with poor nutritional status had less ^137^Cs distributed in their muscle tissue than fish with good nutritional status. The ^137^Cs distribution within fish bodies is potentially useful for assessing human risks through freshwater fish consumption and for estimating dose of the fish themselves.

## Materials and methods

### Study area

The study area was Lake Inba which is located in the northwest part of Chiba Prefecture, Japan (latitude 35° 48′ north and longitude 140° 15′ east) and monthly sampling of fish was done from September 2016 until March 2020. This lake is surrounded by paddy fields, and its water has agricultural, industrial and drinking water uses. This lake is a polymictic lake with a surface area of 11.55 km^2^. The water depth is 1.7 m on average (maximum 2.5 m) and residence time is about 0.08 years^52^. The water depth is controlled by the local government. The lake is about 220 km away from FDNPP. Lake Inba and its surrounding areas were contaminated with fallout radionuclides after the FDNPP accident. According to the report from Ministry of Education, Culture, Sports, Science and Technology (MEXT), deposition densities of radiocesium (^137^Cs + ^134^Cs) in the surrounding environment was from 1.0 × 10^4^ to 3.0 × 10^4^ Bq m^−2^ in September 2011^9^.

### Fish samples and their processing

Eight kinds of freshwater fishes were caught using stake nets placed close to the shore of Lake Inba starting in September 2016. A maximum of 4 nets were set up for one monthly sampling. Each net was put in a separate fixed position from the previous one-day-sampling period. Both surface and bottom fishes were caught at the same time because the lake is shallow. The kinds caught were: snakehead (*Channa argus*), largemouth bass (*Micropterus salmoides*), channel catfish (*Ictalurus punctatus*), bluegill (*Lepomis macrochirus*), common carp (*Cyprinus carpio*), barbel steed (*Hemibarbus barbus*), silver carp (*Hypophthalmichthys molitrix*), and crucian carp (Carassius spp.: *C. auratus langsdorfii* and *C. cuvieri*).

All fish were caught by a researcher who is a member of a local fishery cooperative having license of a class 5 common fishery. The experimental protocols followed the regulations concerning animal experiments of National Institutes for Quantum and Radiological Science and Technology (QST) and were approved by the Institutional Animal Care and Use Ethics Committee of QST. The study was approved by the President of QST. All experiments were performed in accordance with the ARRIVE guidelines (https://www.nc3rs.org.uk/arrive-guidelines) and relevant regulations.

The fish were stored onto the ice cubes in a fishing cooler until they were brought back to the laboratory. In accordance with the Invasive Alien Species Act in Japan (Law No. 78, 2004) largemouth bass, channel catfish, and bluegill were transported after the kill. Total length, body length, body depth, body width, and fresh weight were measured for most fish samples in the laboratory. The condition factor (*K*) of crucian carp was calculated from the following equation [Eq. (1)]:

1 $$K = W/L^{b} \times 100$$

where W is the fresh weight of crucian carp in grams, and L is the body length in centimeter. The *b* value is a factor in the standard allometric equation

2 $$W = aL^{b}$$

and it was determined empirically using data collected in this study (Fig. S1). These fish samples were kept at 4 °C in a laboratory refrigerator until the dissection in the following days.

Fish bodies were washed with a brush and then dissected to separate muscle, bone, internal organs, spawn, milt and the remaining parts including the head, gills, skin fins, and muscle residues. Contents of their digestive organ were removed during the dissection period. However, fish having no stomach, i.e. common carp (*Cyprinus carpio*) and crucian carp (*Carassius* spp*.*: *C. auratus langsdorfii* and *C. cuvieri*), were processed without the removal of undigested contents. Bony parts excluding the head were immersed in 80˚C water until the soft tissues were discolored. After that, tissues attached on bones were careful removed by using a nylon brush. We did not consider loss of ^137^Cs from bone parts by this heating process. Finally, dissected parts were lyophilized. To obtain water contents, the weight of these parts was measured before and after lyophilization. The lyophilized samples were powdered with a grinder (Labo Milser LM-PLUS, Osaka Chemical Co., Ltd.), and the powdered samples were packed into a U8 polypropylene container (D: 55 mm × H: 64 mm, 100 mL) for the analysis of ^137^Cs. The mass of the measured samples ranged from 0.72 to 98 g-dry with an average of 20 g-dry. All parts of an individual fish were combined together and mixed well to determine the total ^137^Cs in whole-body after the measurement of ^137^Cs activity in each part.

### Growth curve of crucian carp

The growth curve of crucian carp was determined from the relationship between age and body length of some specimens. The age of crucian carp was determined with otoliths (ear stones), collected from 62 individuals, by counting annuli on the otolith. Otoliths were removed from head parts and enclosed in a plastic resin. Thin sections were prepared, and otolith rings were counted by Marino Research Co., Ltd. (Mie, Japan). Then, ages of all the captured crucian carp in this study were estimated using the obtained growth curve. These fish samples for age determination were not used for the analysis of the distribution of ^137^Cs because the amount of ^137^Cs in whole-body could not be determined due to the lack of the head part.

The von Bertalanffy growth function (VBGF) was used for modeled fish growth. This model is given by the following equation:

3 $$L \, = \, L_{\infty } \left[ {{1} - {\text{exp }}\left\{ { - k\left( {t - t_{0} } \right)} \right\}} \right]$$

where *L* is the expected body length at age *t*, *L*_*∞*_ is the asymptotic length, *k* is the growth rate coefficient, and *t*_0_ is age at which the fish would have had body length of zero (that is, *t*_0_ is a modeling artifact to adjust the equation for the initial size of the fish). Three parameters, *L*_*∞*_, *k*, and *t*_0_, in VBGF were obtained by non-linear least squares and maximum likelihood estimation. This calculation was carried out by the solver add-in in Microsoft Excel for Microsoft 365. The representative body length of each age was calculated from the obtained model formula.

### Analysis of ^137^Cs activity concentrations

The activity concentrations of ^137^Cs were determined using high-purity germanium detectors (GMX- and GEM-types, ORTEC, SEIKO EG&G Co., Ltd.; GC4018 and GX-4018, CANBERRA Industries Inc.) with Gamma Station (SEIKO EG&G) or Spectrum Explorer software (CANBERRA). The relative efficiencies of the germanium detectors used were 25.0%, 35.0%, 45.9% and ≥ 40.0%, respectively. Each detector was calibrated for energy and detection efficiency with volume radioactivity standard gamma source, MX033U8PP (Japan Radioisotope Association). The measurement accuracy was confirmed using the standard reference material JSAC-047 (Japanese Society for Analytical Chemistry) approximately once a month. Self-absorption correction factors were obtained using a mathematical equation equipped in each software. The measurement times ranged from 10,000 to 227,000 s, and the activity concentrations with relative errors of < 5% were obtained from these measurement times in most cases. The values of measurements were decay-corrected for radioactive decay to the sample collection day. The obtained concentrations on a dry-weight basis were converted to those on a fresh-weight basis with water content values.

### Distribution of ^137^Cs in fish body

Distribution of ^137^Cs in fish body was shown as the percent of the body burden contained in dissected parts using the following equation [Eq. (4)]:

4 $${\text{Distribution }}\;{\text{of}}\;^{{{137}}} {\text{Cs }}\left( \% \right) \, = Q_{{{\text{tissue}}}} /Q_{{\text{whole - body}}} \times { 1}00$$

where *Q*_*tissue*_ is the amount of ^137^Cs in each tissue and organ, and *Q*_*whole-body*_ is that in whole body. The amounts of ^137^Cs were determined as the product of the fresh weights and activity concentrations of ^137^Cs.

### Data analysis

The Bartlett test was conducted to check homogeneity of variance before the analysis of variance. As a result of that test, the Kruskal–Wallis test was adopted to check if the means of several groups were significantly different from each other, unless otherwise noted. When a significant difference was confirmed, differences between multiple group means were explored using Steel–Dwass post hoc comparisons. When the *p*-value of the Bartlett test was more than 0.05, one-way analysis of variance (ANOVA) and the Tukey‒Kramer test were conducted. Regression analysis was carried out to test the strength of the association between the two quantitative variables. These analyses were performed using R software version 3.3.3^53^, but all figures were generated by Microsoft Excel.

## Supplementary information

Supplementary figures.
