# Supplementary material for: Inequality in the distribution of 137Cs contamination within freshwater fish bodies and its affecting factors
Source: Sci Rep. 2021 Mar 11;11:5769. doi: 10.1038/s41598-021-85291-6 (PMC7952404; doi:10.1038/s41598-021-85291-6)
Supplement: Supplementary file 1 — Supplementary figures. [file 41598_2021_85291_MOESM1_ESM.docx]

Inequality in the distribution of ^137^Cs contamination within freshwater fish bodies and its affecting factors

Nobuyoshi ISHII^1*^, Toshio FUROTA^2^, Maiko KAGAMI^2,3^, Keiko TAGAMI^1^, Shigeo UCHIDA^4^.

^1^National Institutes for Quantum and Radiological Science and Technology, Environmental Transfer Parameter Research Group, 4-9-1 Anagawa, Inage-ku, Chiba 263-8555, Japan.

^2^Toho University, Faculty of Science, Department of Environmental Science, 2-2-1 Miyama, Funabashi-shi, Chiba 274-8510, Japan.

^3^Yokohama National University, Graduate School of Environmental and Information Sciences, 79-1 Tokiwadai, Hodogaya-ku, Yokohama 240-8501, Japan.

^4^National Institutes for Quantum and Radiological Science and Technology, Biospheric Assessment for Waste Disposal Team, 4-9-1 Anagawa, Inage-ku, Chiba 263-8555, Japan.

*Corresponding author

Nobuyoshi ISHII

National Institutes for Quantum and Radiological Science and Technology, Environmental Transfer Parameter Research Group, 4-9-1 Anagawa, Inage-ku, Chiba 263-8555 Japan

Tel: +81-43-206-3156

Fax: +81-43-251-4601

e-mail: [ishii.nobuyoshi@qst.go.jp](mailto:ishii.nobuyoshi@qst.go.jp)

Fig. S1 Relationship between body length and fresh weight of crucian carp inhabiting Lake Inba. From this relationship, the b factor in the standard allometric equation *W*=*aL^b^*　(Eq. 2) is set to 3.

Fig. S2 Boxplots showing the proportion of ^137^Cs in the muscle tissues of female crucian carp which were caught each month and assayed. No difference was found for the distribution (n = 134).

Fig. S3 Boxplots showing the proportion of ^137^Cs in the muscle tissues of crucian carp. The population of crucian carp was divided into quarters by their condition factors. Symbols *Q*_1_, *Q*_2_, *Q*_3_, and *Q*_4_ are arranged in the order of increasing condition factor value. The difference in the ^137^Cs distribution was significant (ANOVA, *p*<0.01), and the Tukey‒Kramer post-hoc test showed that only the *Q*_1_ population was significantly different from *Q*_2_, *Q*_3_, and *Q*_4_ populations.
